# Identification of miR-200c and miR141-Mediated lncRNA-mRNA Crosstalks in Muscle-Invasive Bladder Cancer Subtypes

**DOI:** 10.3389/fgene.2018.00422

**Published:** 2018-09-28

**Authors:** Guojun Liu, Zihao Chen, Irina G. Danilova, Mikhail A. Bolkov, Irina A. Tuzankina, Guoqing Liu

**Affiliations:** ^1^Institute of Natural Sciences and Mathematics, Ural Federal University, Yekaterinburg, Russia; ^2^Department of Urology, Nanfang Hospital, Southern Medical University, Guangzhou, China; ^3^Institute of Immunology and Physiology, Ural Branch of the Russian Academy of Sciences, Yekaterinburg, Russia; ^4^School of Life Sciences and Technology, Inner Mongolia University of Science and Technology, Baotou, China

**Keywords:** muscle-invasive bladder cancer, subtypes, miR200c, miR-141, random forest, XGBoost

## Abstract

Basal and luminal subtypes of muscle-invasive bladder cancer (MIBC) have distinct molecular profiles and heterogeneous clinical behaviors. The interactions between mRNAs and lncRNAs, which might be regulated by miRNAs, have crucial roles in many cancers. However, the miRNA-dependent crosstalk between lncRNA and mRNA in specific MIBC subtypes still remains unclear. In this study, we first classified MIBC into two conservative subtypes using miRNA, mRNA and lncRNA expression data derived from The Cancer Genome Atlas. Then we investigated subtype-related biological pathways and evaluated the subtype classification performance using Decision Trees, Random Forest and eXtreme Gradient Boosting (XGBoost). At last, we explored potential miRNA-mediated lncRNA-mRNA crosstalks based on co-expression analysis. Our results show that: (1) the luminal subtype is primarily characterized by upregulation of metabolism-related pathways while the basal subtype is predominantly characterized by upregulation of epithelial-mesenchymal transition, metastasis, and immune system process-related pathways; (2) the XGBoost prediction model is consistently robust for classification of the molecular subtypes of MIBC across four datasets (The area under the ROC curve > 0.9); (3) the expression levels of the molecules in the miR-200c and miR141-mediated lncRNA-mRNA crosstalks differ considerably between the two subtypes and have close relationships with the prognosis of MIBC. The miR-200c and miR-141-dependent mRNA-lncRNA crosstalks might be of great significance in tumorigenesis and tumor progression and may serve as the novel prognostic predictors and classification markers of MIBC subtypes.

## Introduction

Urothelial bladder cancer (UBC) is one of the most common malignant tumors of urinary system. UBC can generally be classified into non-muscle-invasive bladder cancer (NMIBC) and muscle-invasive bladder cancer (MIBC), according to whether the cancer cells are restricted locally in the lamina propria or invade the muscularis propria (Kamat et al., 2016). A great number of studies have reported that according to shared RNA expression patterns or specific genomic alterations MIBC can be further classified into two major subtypes, namely basal and luminal (Sjodahl et al., 2012; Iyer et al., 2013; Choi et al., 2014a,b; Damrauer et al., 2014; Network, 2014; Robertson et al., 2017), which are strikingly similar to the molecular subtypes first described in breast cancer (Perou et al., 2000; Prat et al., 2010). The basal subtype has drawn much attention because it is associated with a more aggressive phenotype and has a higher risk of distant metastasis than luminal subtype (Choi et al., 2014a; Robertson et al., 2017). One reason for the difference is that the two subtypes develop from etiologically different pathways. Pathways that are involved in EMT and immune-associated pathways are upregulated in the basal subtype (Choi et al., 2014a). The molecular biomarkers and pathways involved in MIBC subtypes are the key to understanding its subtype heterogeneity and identifying subtype-specific biomarkers that can be used to better manage MIBC patients.

MicroRNAs (miRNAs) represent one of the most exciting areas of modern medical and biological sciences as they can modulate an immense and complex regulatory network of gene expression in a broad spectrum of developmental and cellular processes, such as cell proliferation, metabolism, apoptosis, and viral infection (Johnston and Hobert, 2003; Hatfield et al., 2005; Zhao et al., 2005; Chen et al., 2006; Oliveira-Carvalho et al., 2012; Huang M. et al., 2016). miRNAs not only have a well-established inhibitory effect on gene expression but also promote gene expression in some cases (Sayed and Abdellatif, 2011; Song et al., 2014), and long non-coding RNAs (lncRNAs) exhibit facilitative or suppressive effects on the gene regulatory network during tumor development (Gontan et al., 2012; Sun et al., 2013). Furthermore, aberration or perturbation in miRNA-mediated mRNA and lncRNA expression levels has a significant correlation with serious clinical consequences, including diseases of diverse origins and malignancy (Salmena et al., 2011; Valinezhad Orang et al., 2014; Tay et al., 2014; Yuan et al., 2014; Zeng et al., 2016; Hu et al., 2017).

Regarding molecular drivers of cancer development, oncogenic mutations and downstream signaling pathways in the pre-cancerous or cancerous cell have been thought to play a crucial role in the cancer formation and progression. In addition, recent studies have shown that metabolic reprogramming plays much more important roles than previously thought in cancer development (Cairns et al., 2011). It is possible that a great number of genomic mutations detected in cancer provide a selective advantage for the cancer cell in the stressful tumor microenvironment by reprogramming cell metabolic processes (Zhang et al., 2015). No matter what is the primary cause of cancer development, it is clear that both the oncogenic signaling and reprogrammed metabolisms involve numerous genes, working in a concerted manner in a complex network. Gene regulatory network-based view can, therefore, provide a deeper insight into the cancer development.

The aim of this study is to identify subtype-specific dysregulated miRNA-mediated mRNA-lncRNA interactions and discover new critical subtype-related genes in MIBC.

## Materials and Methods

### Data Acquisition and Pre-processing

The MIBC RNA-Seq (FPKM) and clinical data were obtained from The Cancer Genome Atlas (TCGA) public data portal^1^, and miRNA-Seq (RPM) data was downloaded from the Broad GDAC Firehose^2^. The gene expression datasets of 403 tumor samples and 19 adjacent normal tissue samples contain 19181 mRNAs, 14376 lncRNAs, and 2588 mature miRNAs. The microarray datasets (GSE32894, GSE13507, and GSE31684) derived from Gene Expression Omnibus (GEO) were used to evaluate the performance of classifiers and verify the prognostic use of marker genes^3^.

### Clustering Analysis and Gene Set Enrichment Analysis

Consensus clustering (Monti et al., 2003) is a method that provides quantitative evidence for determining the number and membership of possible clusters within a dataset, such as RNA-Seq and microarray. For CC analysis, the RPKM gene expression data was pre-processed to detect the most highly expressed and variable genes across samples. We removed 25% genes that have the low arithmetic mean of the given gene across samples. Then the MAD was used to select the most highly expressed and variable 3,000 mRNAs, 300 miRNAs, and 3,000 lncRNAs. CC available in the R package “ConsensusClusterPlus” was performed on 3,000 mRNAs, 300 miRNAs, and 3,000 lncRNAs with 403 tumor samples, using the following key parameters: reps = *50*, innerLinkage = *complete*, clusterAlg = *hc*, k = *6*, and distance = *pearson* (Wilkerson and Hayes, 2010).

Cluster of cluster analysis is a method of integrating the primary clustering results into final cluster assignments. Each sample is represented as a binary vector, whose length is ∑ _i=1_^t^K_i_ (where *t* is the number of datasets and K_i_ is the number of clusters for dataset (*i*), to implement subsequent clustering analysis. We first conducted the CoC analysis on the clustering results of mRNA, miRNA, and lncRNA dataset to obtain a binary dataset. The CC was once more performed on the binary dataset for generating final clusters. Number of final clusters (K) was estimated by commonly used methods including ASW, CPCC, Relative Change in Area under Cumulative density function [^Δ^(*K*)], and PAC (Şenbabaoglu et al., 2014).

In order to explore subtype-associated biological processes, GSEA (Subramanian et al., 2005) was conducted using three gene set datasets (GO-BP, KEGG, and Hallmark gene sets]. The following parameters were taken for GSEA: Number of permutations = *1000*, Permutation type = *gene_set*, Enrichment statistic = *weighted*, Metric for ranking genes = *Signal2Noise*.

### Differentially Expressed Genes and Machine Learning

“Ballgown” (R package) was used to identify DEGs between tumor and normal samples (Frazee et al., 2015). *F*-test was used in “Ballgown”, and DEGs here were defined as those with FDR adjusted *p*-value < 0.05 (Benjamini–Hochberg method) and |log_2_fold change| > 0.57).

Three tree-based machine learning methods, namely DTs, RF, and eXtreme Gradient Boosting (XGBoost or XG), were performed on 3000 mRNAs, 300 miRNAs, and 3000 lncRNAs for MIBC subtype classification. The area under the ROC curve (AUC) was used to estimate the performance of the classification methods. For each classification method, MIBC samples were randomly divided into training (60%) and testing (40%). We performed RF with different parameter values of *ntree* and *mtry*, and used 10-fold cross-validation to acquire the mean accuracy. XGBoost was implemented with the following parameters: gamma = *1*, min_child_weight = *1*, max_depth = *14*, nrounds = *2000*. In order to optimize the parameter *iter* (number of iterations) of XGBoost, we obtained 10-fold cross-validation performance for each *iter* and selected the *iter* value that generated the best performance. For DTs, the following parameters were taken: minCases = 20 and CF = 0.25. Moreover, the well-performed classifiers in this study were trained on the TCGA-derived RNA expression data and were tested on the GSE32894 to further evaluate their performance. All machine learning methods were implemented using R packages including “C5.0”, “randomForest”, and “XGBoost” packages (Liaw and Wiener, 2002; Chen and Guestrin, 2016; Kuhn et al., 2018).

The overlap between the feature genes obtained by the well-performed classifiers and DEGs was referred to as DEFGs. GO enrichment analysis available in the R package “clusterProfiler” was performed on DEFGs to identify their enriched GO terms (Yu et al., 2012). A multiple-test correction was done using the method proposed by Benjamini and Hochberg, in which an adjusted *p*-value < 0.05 was considered to represent statistical significance.

### Construction of a Subtype-Related mRNA-miRNA-lncRNA Network

Pairwise Pearson’s correlation analysis was carried out on the DEFGs. The lncRNA-miRNA pairs, miRNA-mRNA pairs, and lncRNA-mRNA pairs with |r| > = 0.4 and *p*-value < 0.05 were considered to be co-expressed gene pairs. If both elements in a co-expressed lncRNA-mRNA pair are simultaneously co-expressed with a miRNA, it is defined as a miRNA-dependent lncRNA-mRNA co-expressed interaction. A miRNA-dependent lncRNA-mRNA network was established using Cytoscape software (version 3.5.1). miRWalk2.0 (Dweep et al., 2011) is an integration of six widely used databases (miRWalk, miRanda, miRDB, miRNAMap, RNA22, and Targetscan) and supplies the biggest available collection of predicted and experimentally verified miRNA-target interactions. Our inferred co-expressed interactions including mRNA-miRNA and lncRNA-miRNA interactions were compared to those derived from miRWalk2.0. An mRNA is considered to be a true target of miRNA if their interaction occurs in at least four databases, and an lncRNA is considered to be a true target of miRNA if their interaction is supported in at least one database among miRWalk, miRanda, and Targetscan.

### Survival Analysis

We further assessed whether the genes in the inferred interactions are correlated with the overall survival of MIBC patients. Based on the mean expression level of the genes, patient samples were divided into high and low expression groups. We performed survival analysis available in R package “survival” (Therneau, 2015) using the Kaplan–Meier curve (K–M curve) method. A log-rank test was used to compare survival times between two groups, and *p* < 0.05 was considered to represent the statistical significance.

## Results

### Clustering Analysis and GSEA

**FIGURE 1:**
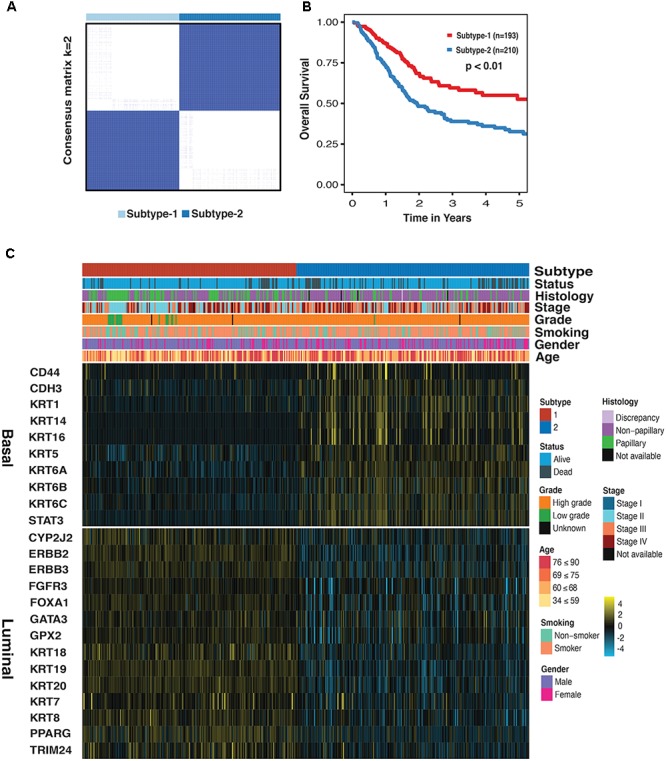
Subtype-1 and subtype-2 Classification for MIBC. **(A)** Hierarchically clustered heatmap for CoC dataset (*K* = 2). **(B)** A K-M plot for overall 5-year survival of subtype-1 and subtype-2 (subtype-1 = 193, subtype-2 = 210, *p* < 0.01). **(C)** Heatmap depicts the expression profiles of basal (up) and luminal (down) biomarkers in subtype-1 and subtype-2. Covariate annotation tracks show selected clinical features. The yellow and turquoise colors correspond to high and low relative expression, respectively.

We first performed the CC on mRNA, miRNA, and lncRNA expression datasets to obtain the clustering results. By applying the CoC analysis to the clustering outcomes of CC, a binary dataset was obtained, which was referred to as CoC dataset. The CC was once again performed on the CoC dataset to generate the different Ks, and the ASW, CPCC, Δ*K*, and PAC were used to evaluate the optimal K (**Supplementary Figure S1**). As a result, for the CoC dataset, ASW evaluation suggests the optimal K of 6 and CPCC, Δ*K*, and PAC indicate the optimal *K* of 2. Given that *K* = 2 is the consistent optimal value, we chose *K* = 2 as a solution, dividing MIBC samples into two subtypes, namely subtype-1 and subtype-2. The hierarchically clustered heatmap of *K* = 2 for CoC dataset was shown in **Figure 1A**. Survival curves regarding two subtypes were plotted using the K-M method. Our results have shown that 5-year overall survival rate with regard to subtype-1 is 55% and 30% for subtype-2, indicating that they differ considerably in clinical prognosis (**Figure 1B**, *p* < 0.01). The heatmap depicting basal biomarkers, luminal biomarkers, and clinical indicators for the two subtypes was shown in the **Figure 1C**. The subtype-1 is characterized by the high expression of luminal markers such as CYP2J2, ERBB2, and KRT18, while the subtype 2 is characterized by high expression of basal markers such as CD44, CDH3, and KRT1. The Pearson’s chi-squared test is utilized to compare clinical indicators between the two subtypes. The histology, stage, grade, and status are significantly different between the two subtypes, and gender almost differs between the two subtypes (**Supplementary Table S1**). The subtype-1 and subtype-2 resemble the luminal and basal subtype, respectively, in terms of K–M curves, biomarkers, and clinical indicators, therefore, which were redefined as luminal and basal subtypes (Choi et al., 2014a).

Gene set enrichment analysis was done for the basal and luminal subtypes, and the results were shown in **Tables 1**, **2**. Upregulated pathways in luminal subtype are mainly involved in metabolism (e.g., oxidative phosphorylation, cytochrome P450, and fatty acid metabolism) (**Table 1**). Whereas, upregulated pathways in the basal subtype are principally related to immune system process (e.g., extracellular structure organization, allograft rejection, mTORC1 signaling, and TNF-a signaling via NF-kB), metastasis, and EMT (**Table 2**).

**Table 1:** Top-ranked terms of GO-BP, KEGG and Hallmark gene sets for the luminal subtype.

Gene set name	Size	NES	FDR *q*-value
**GO-BP**			
GO monocarboxylic acid catabolic process	95	2.5151	0
GO oxidative phosphorylation	73	2.4333	0
GO fatty acid catabolic process	80	2.4284	0
GO fatty acid beta oxidation	51	2.3211	0
GO electron transport chain	78	2.3116	0
GO organic acid catabolic process	92	2.2781	2.26E-04
GO mitochondrial respiratory chain complex assembly	42	2.1788	0.0014
GO lipid oxidation	63	2.1586	0.0015
GO mitochondrial respiratory chain complex i biogenesis	199	2.1449	0.0018
GO establishment of protein localization to endoplasmic reticulum	70	2.1285	0.0022
**KEGG**			
KEGG ribosome	87	2.3289	0
KEGG alpha linolenic acid metabolism	19	2.0770	5.29E-04
KEGG metabolism of xenobiotics by cytochrome p450	68	2.0424	5.36E-04
KEGG valine leucine and isoleucine degradation	44	1.9767	0.00137
KEGG drug metabolism cytochrome p450	70	1.9727	0.00120
KEGG oxidative phosphorylation	116	1.9372	0.00209
KEGG peroxisome	78	1.9320	0.00214
KEGG fatty acid metabolism	42	1.9184	0.00229
KEGG retinol metabolism	63	1.8697	0.00391
KEGG linoleic acid metabolism	29	1.8393	0.00482
**Hallmark gene sets**			
Hallmark oxidative phosphorylation	198	1.5145	0.05830
Hallmark bile acid metabolism	112	1.4110	0.07668
Hallmark peroxisome	103	1.4095	0.05174
Hallmark adipogenesis	191	1.3794	0.05125
Hallmark fatty acid metabolism	156	1.2596	0.11892


*NES, normalized enrichment score; GO-BP, Gene Ontology Biological Process; KEGG, Kyoto Encyclopedia of Genes and Genomes. Size is the number of genes in the gene set. A positive NES means that genes over-represented in the gene set are upregulated in luminal subtypes.*

**Table 2:** Top-ranked categories of GO-BP, KEGG and Hallmark gene sets for the basal subtype.

Gene set name	Size	NES	FDR *q*-value
**GO-BP**			
GO extracellular structure organization	297	–2.8256	0
GO antigen processing and presentation of exogenous peptide antigen via mhc class i	65	–2.7258	0
GO antigen processing and presentation	206	–2.6334	0
GO antigen processing and presentation of peptide antigen	170	–2.6246	0
GO antigen processing and presentation of peptide antigen via mhc class i	90	–2.6134	0
GO chondroitin sulfate biosynthetic process	25	–2.6008	0
GO collagen fibril organization	36	–2.5958	0
GO regulation of innate immune response	349	–2.5825	0
GO positive regulation of defense response	360	–2.5802	0
GO cytokine mediated signaling pathway	440	–2.5675	0
**KEGG**			
KEGG focal adhesion	197	–2.6862	0
KEGG cytokine cytokine receptor interaction	257	–2.5127	0
KEGG ecm receptor interaction	84	–2.512	0
KEGG proteasome	43	–2.4802	0
KEGG leishmania infection	69	–2.4718	0
KEGG viral myocarditis	68	–2.4178	0
KEGG hematopoietic cell lineage	85	–2.4134	0
KEGG regulation of actin cytoskeleton	211	–2.3911	0
KEGG allograft rejection	35	–2.3902	0
KEGG autoimmune thyroid disease	50	–2.3778	0
**Hallmark gene sets**			
Hallmark epithelial-mesenchymal transition	197	–3.2473	0
Hallmark inflammatory response	197	–3.0190	0
Hallmark interferon gamma response	197	–2.9964	0
Hallmark interferon alpha response	94	–2.9491	0
Hallmark allograft rejection	199	–2.9010	0
Hallmark G2M checkpoint	194	–2.6389	0
Hallmark E2F targets	196	–2.6177	0
Hallmark TNF-a signaling via NF-kB	198	–2.5512	0
Hallmark complement	195	–2.5475	0
Hallmark mTORC1 signaling	198	–2.441	0


*All abbreviations are the same as in **Table 1**. A negative NES value indicates that genes over-represented in the gene set are upregulated in the basal subtype.*

### Differentially Expressed Genes and Machine Learning

The DEGs that could distinguish tumor from normal samples were analyzed and visualized as volcano plots (**Supplementary Figures S2A–C**). In total, 208 miRNAs (148 upregulated and 60 downregulated), 2488 lncRNAs (1402 upregulated and 1086 down-regulated), and 4167 mRNAs (2314 upregulated and 1853 downregulated) are differentially expressed.

**FIGURE 2:**
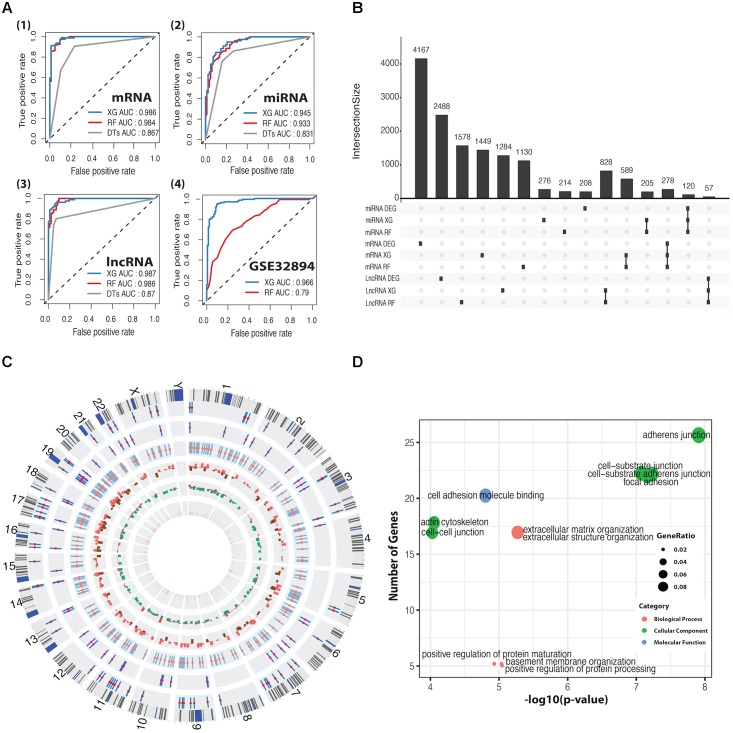
ROC curves for classification predictors and information for differentially expressed feature genes (DEFGs). **(A)** ROC curves for XGBoost, RF, and DTs classifiers in mRNA, miRNA, lncRNA, and GSE32894 dataset. **(B)** An UpSet plot for intersected information between DEGs and the feature genes obtained by XGBoost and RF. **(C)** A Circos plot for 278 DEFmRNAs with gene symbols, chromosomal cytobands, and other information. From inside to outside, variable weight from RF (brown), fold change from DEGs (up in red, down in green), *p*-value from univariate Cox’s proportional regression analysis (*p* > 0.05 in red, *p* < 0.05 in brown), DEFlncRNAs (coral), DEFmiRNAs (deep pink), DEFmRNAs (red). **(D)** Bubble plots for enriched GO terms generated from 278 DEFmRNAs. The *x*-axis represents the –log_10_(*p*-value) of each term and the *y*-axis represents the number of genes in each term.

We applied DTs, RF, and XGBoost for the basal and luminal subtype classification based on mRNA, miRNA, and lncRNA expression dataset, and AUC was used to evaluate their performance. As shown in **Figure 2A**, XGBoost outperforms RF and DTs, having AUC values of 98.6, 94.5, and 98.7%, respectively, in mRNA, miRNA and lncRNA-based classification. Details regarding 10-fold cross-validation procedure can be found in **Supplementary Figure S3**. DTs was excluded in the following comparison, as it is significantly inferior to RF and XG on average. By using the CC method, the GSE32894 dataset containing 28 biomarkers and 190 samples was grouped into two subtypes prepared for the classification task. The heatmap plots and the K–M curves for the two subtypes were shown in **Supplementary Figure S4**. We trained the well-performed classifiers (RF and XG) on mRNA dataset that was derived from TCGA and tested them on GSE32894 dataset. The results demonstrated that XGBoost has a better performance than RF (**Figure 2A4**).

The intersection between DEGs and feature genes obtained by RF and XG was defined as DEFGs, which includes 57 lncRNAs, 120 miRNAs, and 278 mRNAs. The Upset plot and heatmap plots for DEFGs were shown in **Figure 2B** and **Supplementary Figures S2D–F**. The genetic and clinical information of DEFGs was visualized in **Figure 2C**. GO enrichment analysis indicated that differentially expressed feature mRNAs are enriched with adherens junction, cell-substrate junction, cell-cell junction, cell-substrate adherens junction, and focal adhesion (**Figure 2D**). These GO terms have been found to play roles in tumorigenesis and tumor progression by regulating T-cell signaling, innate immunity, TGF-β signaling, and Wnt signaling through post-translational modification (Kikuchi et al., 2006; Lönn, 2010; Liu et al., 2016; Cho et al., 2018; Kuwabara et al., 2018).

### Construction of Subtype-Related mRNA-miRNA-lncRNA Network

**FIGURE 3:**
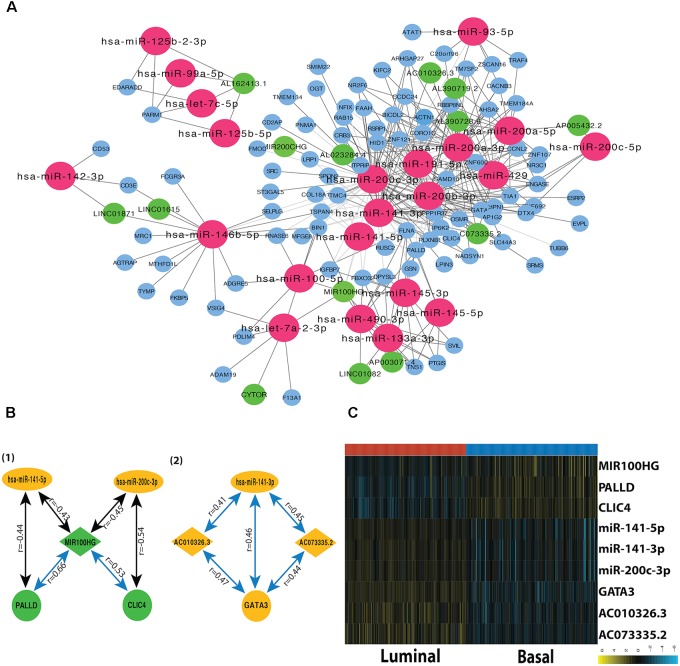
Subtype-related miRNA-dependent mRNA-miRNA interactions. **(A)** The co-expression network for miRNA-mediated mRNA-lncRNA interactions. The crimson nodes represent miRNAs, the green nodes represent lncRNAs, and the sky blue nodes represent mRNAs. **(B)** miRNA-mediated mRNA-lncRNA interactions validated by mirWalk 2.0. The green color represents downregulation in tumor compared with normal sample whereas the yellow color corresponds to upregulation in the tumor. The blue lines represent positive correlations and black lines represent negative correlations. **(C)** The heatmap depicts the expression level of nine DEFGs in basal and luminal subtypes. The yellow and turquoise colors correspond to high and low relative expression, respectively. Original expression value was log2 transformed.

A miRNA-dependent mRNA-lncRNA co-expression network was constructed, which consists of 90 mRNAs, 22 miRNAs, and 14 lncRNAs (**Figure 3A**). The miRNA-dependent mRNA-lncRNA crosstalks verified in miRWalk database contain four miRNA-mediated mRNA-lncRNA interactions (**Figure 3B**). To be specific, two co-expressed lncRNA-mRNA pairs, AC010326.3-GATA3 and AC073335.2-GATA3, are positively regulated by miR-141-3p; The lncRNA-mRNA pairs, such as MIR100HG–CLIC4 and MIR100HG–PALLD, are negatively regulated by miR-200c-3p and miR-141-5p, respectively. All the nine genes in the network differ in their expression between the two subtypes (**Figure 3C**). For instance, as compared to the luminal subtype, the basal subtype is characterized by a lower expression level of six genes (miR-200c-3p, miR-141-3p, miR-141-5p, GATA3, AC010326.3, and AC073335.2) and a higher expression level of the other three genes (MIR100HG, PALLD, and CLIC4), suggesting that all the nine genes can be used as potential markers for the two MIBC subtypes. In addition, GO analysis showed that the mRNAs in the network (CLIC4, PALLD, and GATA3) are related to cytoskeleton.

### Survival Analysis of Crosstalk-Involved Genes

**FIGURE 4:**
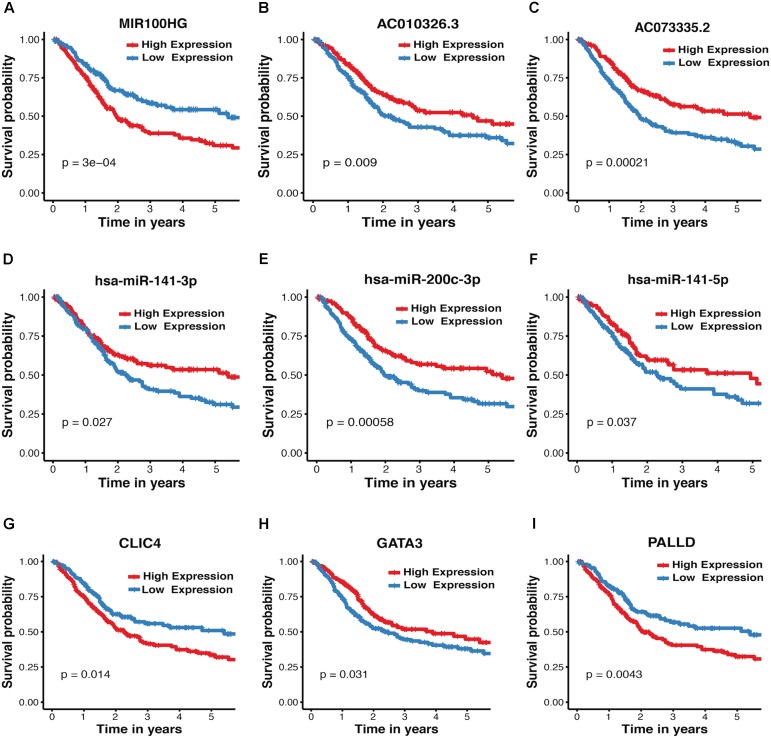
Kaplan–Meier plots for crosstalk-involved genes. **(A–I)** The red lines represent the high expression of crosstalk-involved genes while blue lines represent the corresponding low expression. The *p*-value was calculated using a log-rank test, where *p* < 0.05 represents statistical significance.

The association between expression levels of crosstalk-involved genes and MIBC prognosis was analyzed by K–M method. Strikingly, the results revealed that all of them are closely related to prognosis of MIBC. Specifically, the higher expression level of miR-141-5p, miR-141-3p, AC010326.3, AC073335.2, miR-200c-3p, and GATA3 predicts better prognosis, indicating that they may function as tumor suppressors (**Figures 4B–F,H**); In contrast, the higher expression level of MIR100HG, PALLD, and CLIC4 is associated with worse prognosis, suggesting that they may play an oncogenic role (**Figures 4A,G,I**). In addition, the association between MIBC prognosis and expression levels of crosstalk-related mRNAs (CLIC4, PALLD, and GATA3) was validated in two independent microarray datasets (GSE13507 and GSE31684), suggesting again their prognosis value in MIBC (**Supplementary Figure S5**).

## Discussion

In this study, we have investigated miRNA-dependent mRNA-lncRNA interactions in MIBC basal and luminal subtypes using bioinformatics approaches. On the basis of MIBC mRNA, miRNA, and lncRNA expression datasets obtained from TCGA, 403 MIBC samples were reliably classified into two intrinsic molecular types, which resemble basal and luminal subtypes identified previously (Choi et al., 2014a). A number of subtype-related pathways were identified through GSEA. Moreover, we conducted and compared subtype classification performance among tree-based machine learning algorithms, and found XGBoost outperforms other classifiers. Additionally, we implemented a gene co-expression analysis on DEFGs and successfully identified subtype-specific mRNA-lncRNA crosstalks, which differ considerably between basal and luminal subtypes and have close relationships with the prognosis of MIBC.

Subtype-related pathways presented in this study (**Tables 1**, **2**) are largely consistent with the previously identified (Choi et al., 2014a; Hurst and Knowles, 2014; McConkey et al., 2016; Ochoa et al., 2016; Hau et al., 2017; Seiler et al., 2017; Baker et al., 2018). In general, pathways that are involved in the EMT, metastasis, and immune system process, are upregulated in the basal subtype, whereas, metabolic-related pathways are upregulated in the luminal subtype. Th pathways enriched in basal and luminal subtypes provide a biological explanation for their distinctively different clinical and pathological behaviors. However, the mechanisms by which some other pathways shown in our results, like valine leucine, isoleucine degradation, autoimmune thyroid disease, hematopoietic cell lineage and viral myocarditis, play a role in MIBC subtypes deserve further investigation.

Many machine learning methods have been broadly applied in many areas of biology such as gene family classification, hepatotoxicity prediction, RNA methylation prediction, cancer prediction and classification (Zou et al., 2014; Kourou et al., 2015; Liao et al., 2017, 2018; Su et al., 2018; Wei et al., 2018a,b). As suggested in previous studies, RF is a powerful classifier for classifying gene expression data (Wu et al., 2003; Lee et al., 2005; Ishwaran et al., 2010). And XGBoost keeps winning in “every” Kaggle competition and has become a really popular tool among data scientists (Ren et al., 2017; Torlay et al., 2017; Zhang and Zhan, 2017). Recently, XGBoost has been successfully applied to many classification problems, such as pan-cancer classification (Li et al., 2017) and prediction of RNA-protein interactions (Jain et al., 2018). However, no comparison between RF and XGBoost in terms of cancer classification has been made in the past. In this study, we compared the performance of DTs, RF, XGBoost in classifying basal and luminal subtypes. Our results clearly demonstrated the advantage of XGBoost in gene expression data-based cancer classification (**Figure 2A**).

Previous studies investigated MIBC-associated miRNAs and their target genes without considering the genetic heterogeneity of MIBC subtypes (Martens-Uzunova et al., 2014; Huang T. et al., 2016; Xue et al., 2016; Zhong et al., 2016). It is therefore important to elucidate the subtype-related molecular pathways and identify novel biomarkers for MIBC subtypes. In this study, we systematically explored MIBC subtype-related gene co-expression networks. A total of three mRNAs (GATA3, CLIC4, and PALLD), three miRNAs (miR-200c-3p, miR-141-3p, and miR-141-5p), and three lncRNAs (AC010326.3, AC073335.2, and MIR100HG) were found in miRNA-mediated mRNA-lncRNA crosstalks, which differ considerably in their expression between basal and luminal subtypes (**Figure 3**), and their expression level is significantly associated with the prognosis of MIBC (**Figure 4**). It was previously observed that miR-141-5p, miR-141-3p, miR-200c-3p, and GATA3 are the most important markers of luminal subtype, which is consistent with our results (Robertson et al., 2017). Besides, previous studies found that the down-regulation of miR-200c and miR-141 is associated with elevated ZEB1 (Wiklund et al., 2011; Shan et al., 2013; Mahdavinezhad et al., 2015), and the down-regulation of miR-200c is also coupled with the down-regulation of BMI-1 and E2F3 (Liu et al., 2014), which play an important role in the invasion, migration, and EMT of bladder cancer.

It has been shown that some other genes in the crosstalk are also closely related to cancer. For example, AC073335.2, a highly expressed lncRNA in human glioblastoma, is involved in tumorigenesis via acting as a competing endogenous RNA of miR-940 (Shi et al., 2017). MIR100HG was previously reported to act as a regulator of hematopoiesis and oncogenes in many cancers (Emmrich et al., 2014; Nair, 2016; Shang et al., 2016; Wieczorek and Reszka, 2018; Zhang et al., 2018). In agreement with our findings, MIR100HG was reported to be down-regulated in MIBC and may serve as a significant biomarker for MIBC (Wang et al., 2016). As reported previously, GATA3 is a prognostic marker and inhibits cell migration and invasion in MIBC (Miyamoto et al., 2012; Choi et al., 2014a,b). And, GATA3 is differentially expressed between basal and luminal subtypes and can be used as a luminal-infiltrated marker (Robertson et al., 2017). CLIC4 has a complicated role in cancer. For instance, it functions as a tumor suppressor in lung adenocarcinomas (Okudela et al., 2014). And it promotes the metastasis and development of colorectal cancer (Deng et al., 2014; Peretti et al., 2015). Previous studies have established that the expression of CLIC4 in MIBC has a subtype-dependent pattern (Robertson et al., 2017). And the overexpression of CLIC4 in stroma increases cell migration and invasion and promotes epithelial to mesenchymal transition in multiple human cancers (Shukla et al., 2014). PALLD SNPs were reported to be a significant predictor of prostate cancer-specific mortality (Bao et al., 2011). Our findings are largely consistent with previously reported results, suggesting crosstalk-implicated genes might be of great significance in MIBC pathogenesis and post-transcriptional gene regulation.

The combination of bioinformatics and several machine learning approaches in this study have achieved reliable results regarding the MIBC subtype classification, subtype-associated pathways, and the network-associated markers for MIBC subtypes. The subtype-related genes can not only be used for subtype classification but also serve as a good predictor of cancer prognosis. It is worth noting that we can enhance our study in the following aspects in the future: (1) the crosstalks discovered through computational analyses need to be verified by biological experiments. (2) DEFGs were defined as the overlap between DEGs and feature genes that were determined by XGBoost based on the ranking approximates of Information Gain. This procedure may result in the missing of some highly correlated genes that are also biologically important.

## Conclusion

By conducting bioinformatics analyses, we identified two subtypes of MIBC and lncRNA-mRNA crosstalks mediated by miR-200c and miR-141, which are found to be significantly associated with prognosis, formation, and metastasis of bladder cancer. Our results should be informative for molecular subtype classification, prognosis and molecule-targeted therapy of bladder cancer.

## Author Contributions

GjL and ZC performed the computations. MB and IT contributed to data preparation and analysis. GjL and GqL wrote the manuscript. GqL and ID conceived and designed the study.

## Conflict of Interest Statement

The authors declare that the research was conducted in the absence of any commercial or financial relationships that could be construed as a potential conflict of interest.

## Footnotes

1 https://cancergenome.nih.gov

2 https://gdac.broadinstitute.org

3 https://www.ncbi.nlm.nih.gov

## Supplementary Material

FIGURE S1 The graphs show the evaluation output of ACW, CPCC, Δ*K*, and PAC. CoC datasets represented by green line were used as the criteria to infer optimal K. **(A)** ASW allows us to inference the optimal K by high ASW. **(B)** The optimal K according to CPCC is that the magnitude of CPCC should be very close to one. **(C)** The optimal K according to Δ*K* is the *K* value before the ‘elbow’ or the K where D(K) reaches its maximum. **(D)** PAC allows us to inference the optimal K by the lowest PAC.

FIGURE S2 Volcano plots for DEGs and heatmap plots for DEFGs. **(A–C)** Volcano plots for differentially expressed 4167 mRNAs, 208 miRNAs, and 2488 lncRNAs between tumor and normal samples (adjusted *p*-value < 0.05 and |log_2_fold change| > 0.57). **(D–F)** Heatmap plots for 278 DEFmRNAs, 120 DEFmiRNAs, and 57 DEFlncRNAs. Basal, luminal, and normal samples are represented by the red, blue, and yellow bar, respectively.

FIGURE S3 Parameter selection and Performance of RF and XG in mRNA, miRNA and lncRNA dataset. **(A)** The *x*-axis represents the number of mtry set for RF classifier (1, 5, 10, 15, 20, 25). The *y*-axis represents the corresponding AUC. **(B)** The *x*-axis represents the number of ntree set for RF (20, 400, 600, 800). The *y*-axis represents corresponding obb error rates. The colors correspond to mtry numbers. **(C)** The x-axis represents the number of fold set for RF. The *y*-axis represents corresponding accuracy. The red color shows mean accuracy. **(D)** The *x*-axis represents the number of *iter* set for XG (1, 400, 800, 1200, 1600, 2000, 2400) and the y-axis represents the corresponding accuracy.

FIGURE S4 Heatmap and K–M plots for basal and luminal subtypes of GSE32894. **(A)** Heatmap depicts the expression profiles of basal (up) and luminal (down) biomarkers in GSE32894. The yellow and turquoise color corresponds to high and low relative expression, respectively. **B**. A K-M plot for the overall 5-year survival of basal and luminal subtypes (basal = 52, luminal = 62, *p* < 0.01).

FIGURE S5 Kaplan-Meier plots for CLIC4, PALLD, and GATA3 in GSE13507 and GSE31684. **(A–C)** K–M survival curves showing overall survival according to high expression and low expression of CLIC4, PALLD, and GATA3 in GSE13507. **(D–F)** K–M survival curves showing overall survival according to high expression and low expression of CLIC4, PALLD, GATA3, and MIR100HG in GSE31684.

## Abbreviations

ASW: Average of Silhouette Width
CC: cellular component
CC: consensus clustering
CDF: cumulative density function
CoC: cluster of cluster
CPCC: cophenetic correlation coefficient
DEFGs: differentially expressed feature genes
DEGs: differentially expressed genes
DTs: decision trees
EMT: epithelial-mesenchymal transition
FDR: False Discovery Rate
GO-BP: Gene Ontology Biological Process
GSEA: gene set enrichment analysis
KEGG: Kyoto Encyclopedia of Genes and Genomes
K–M curve: Kaplan–Meier curve
MAD: median absolute deviation
MF: molecular function
MIBC: muscle-invasive bladder cancer
NES: normalized enrichment scores
PAC: proportion of ambiguous clustering
RF: random forest
ROC: receiver operating characteristics curve
XGBoost: eXtreme Gradient Boosting
