# Supplementary figures and images for: Identification of miR-200c and miR141-Mediated lncRNA-mRNA Crosstalks in Muscle-Invasive Bladder Cancer Subtypes
Source: Front Genet. 2018 Sep 28;9:422. doi: 10.3389/fgene.2018.00422 (PMC6172409; doi:10.3389/fgene.2018.00422)

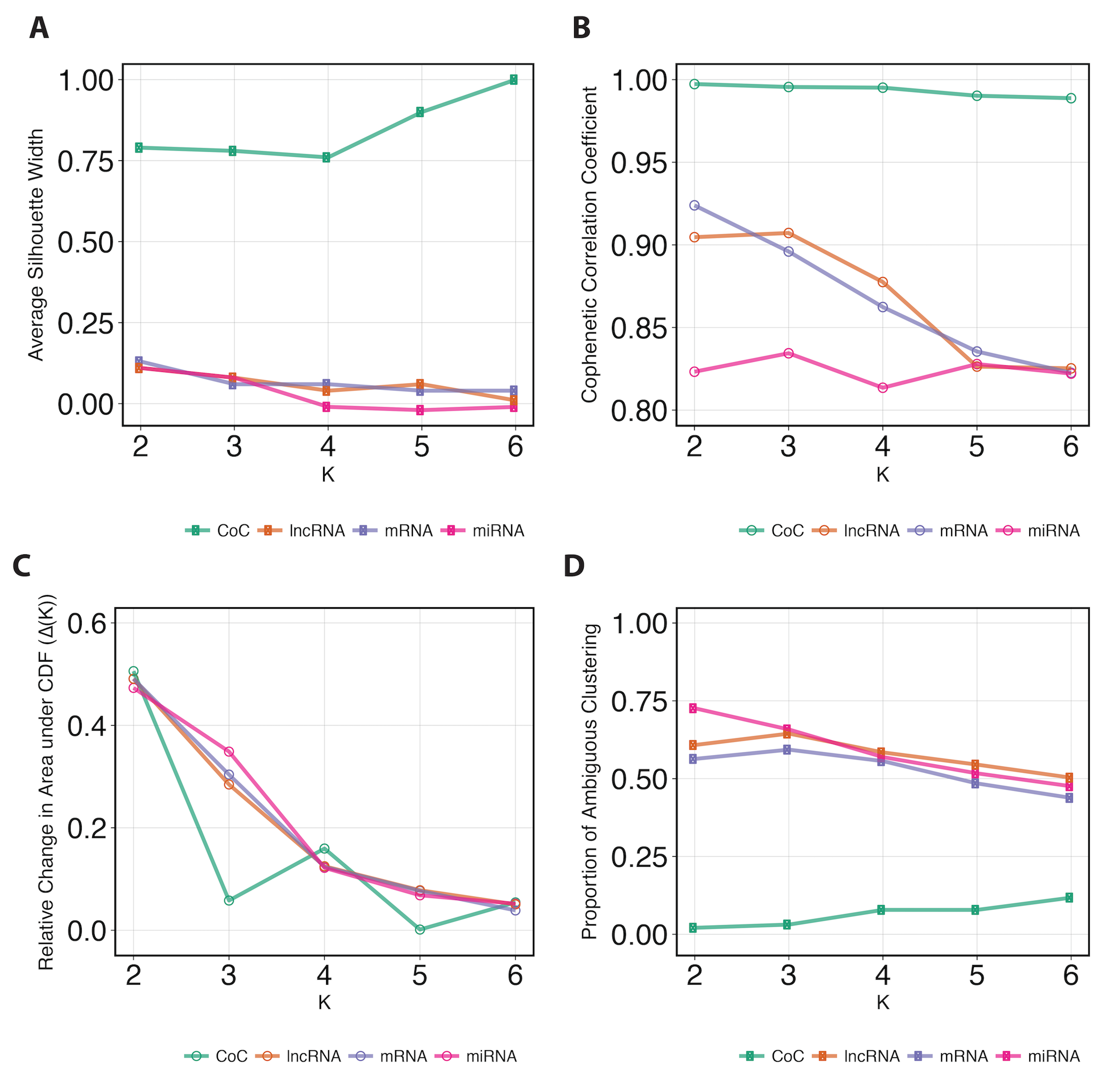

Supplement: FIGURE S1 — The graphs show the evaluation output of ACW, CPCC, ΔK, and PAC. CoC datasets represented by green line were used as the criteria to infer optimal K. (A) ASW allows us to inference the optimal K by high ASW. (B) The optimal K according to CPCC is that the magnitude of CPCC should be very close to one. (C) The optimal K according to ΔK is the K value before the ‘elbow’ or the K where D(K) reaches its maximum. (D) PAC allows us to inference the optimal K by the lowest PAC. [file Image_1.TIF]

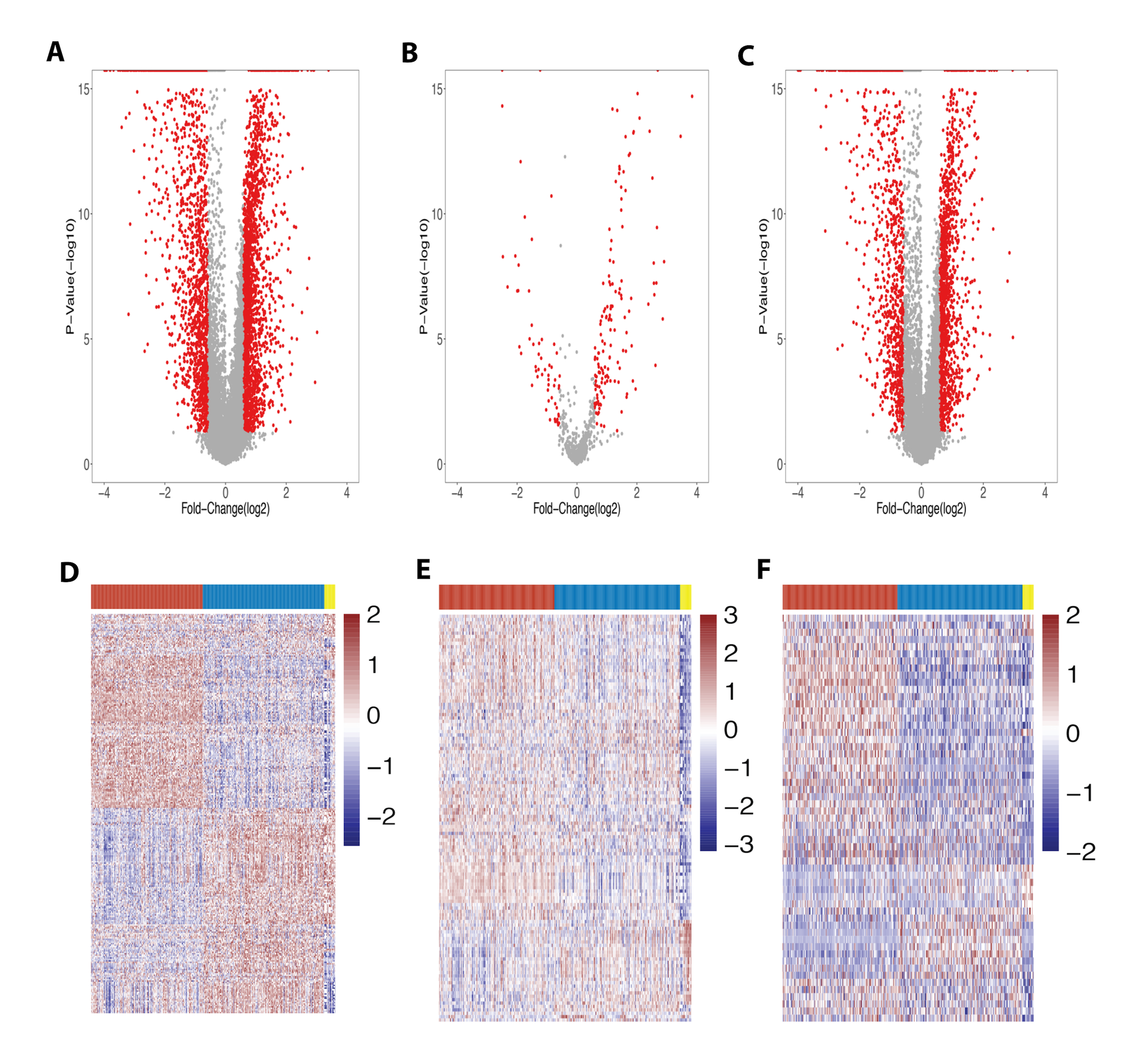

Supplement: FIGURE S2 — Volcano plots for DEGs and heatmap plots for DEFGs. (A–C) Volcano plots for differentially expressed 4167 mRNAs, 208 miRNAs, and 2488 lncRNAs between tumor and normal samples (adjusted p-value < 0.05 and |log2fold change| > 0.57). (D–F) Heatmap plots for 278 DEFmRNAs, 120 DEFmiRNAs, and 57 DEFlncRNAs. Basal, luminal, and normal samples are represented by the red, blue, and yellow bar, respectively. [file Image_2.TIF]

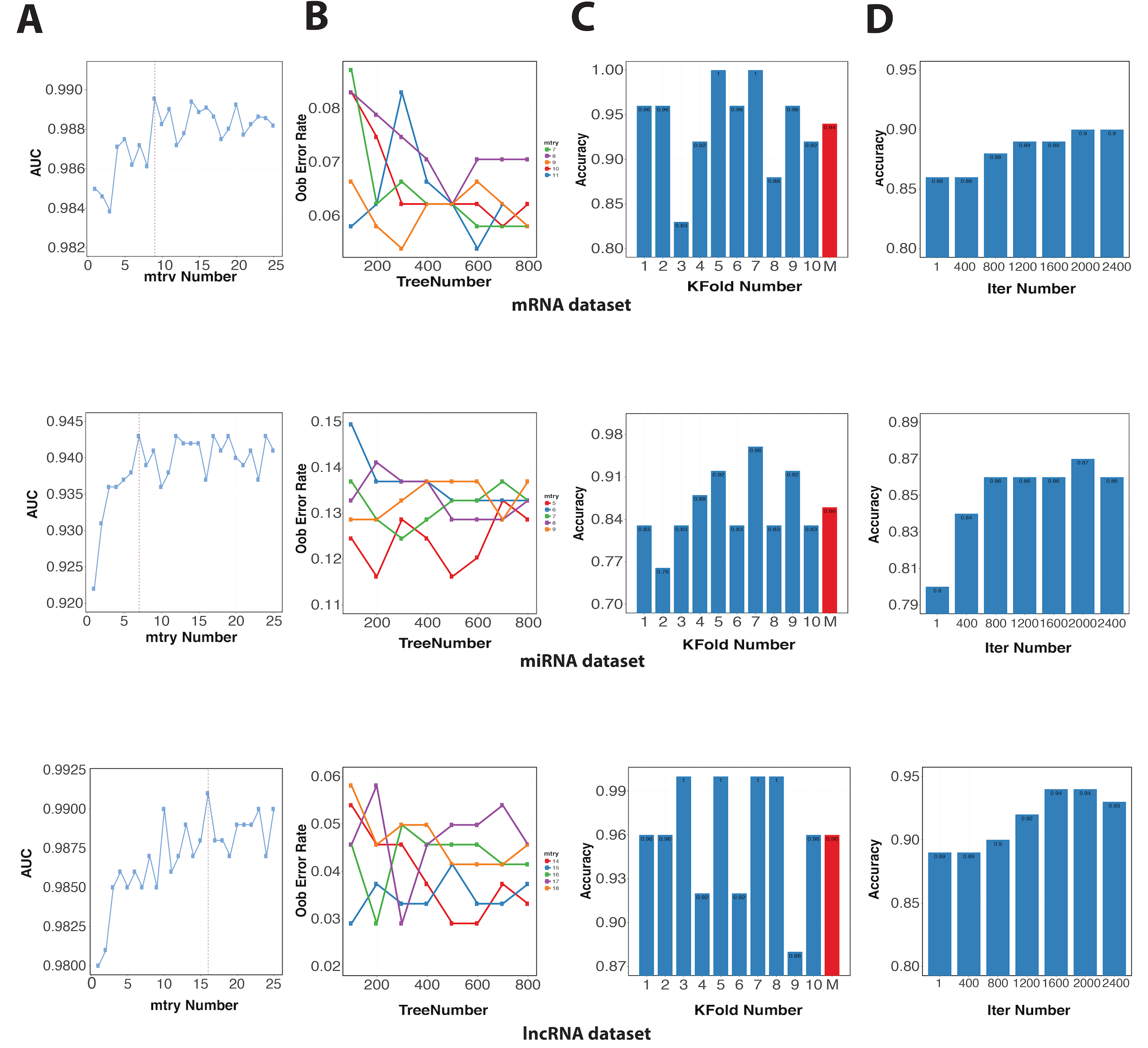

Supplement: FIGURE S3 — Parameter selection and Performance of RF and XG in mRNA, miRNA and lncRNA dataset. (A) The x-axis represents the number of mtry set for RF classifier (1, 5, 10, 15, 20, 25). The y-axis represents the corresponding AUC. (B) The x-axis represents the number of ntree set for RF (20, 400, 600, 800). The y-axis represents corresponding obb error rates. The colors correspond to mtry numbers. (C) The x-axis represents the number of fold set for RF. The y-axis represents corresponding accuracy. The red color shows mean accuracy. (D) The x-axis represents the number of iter set for XG (1, 400, 800, 1200, 1600, 2000, 2400) and the y-axis represents the corresponding accuracy. [file Image_3.TIF]

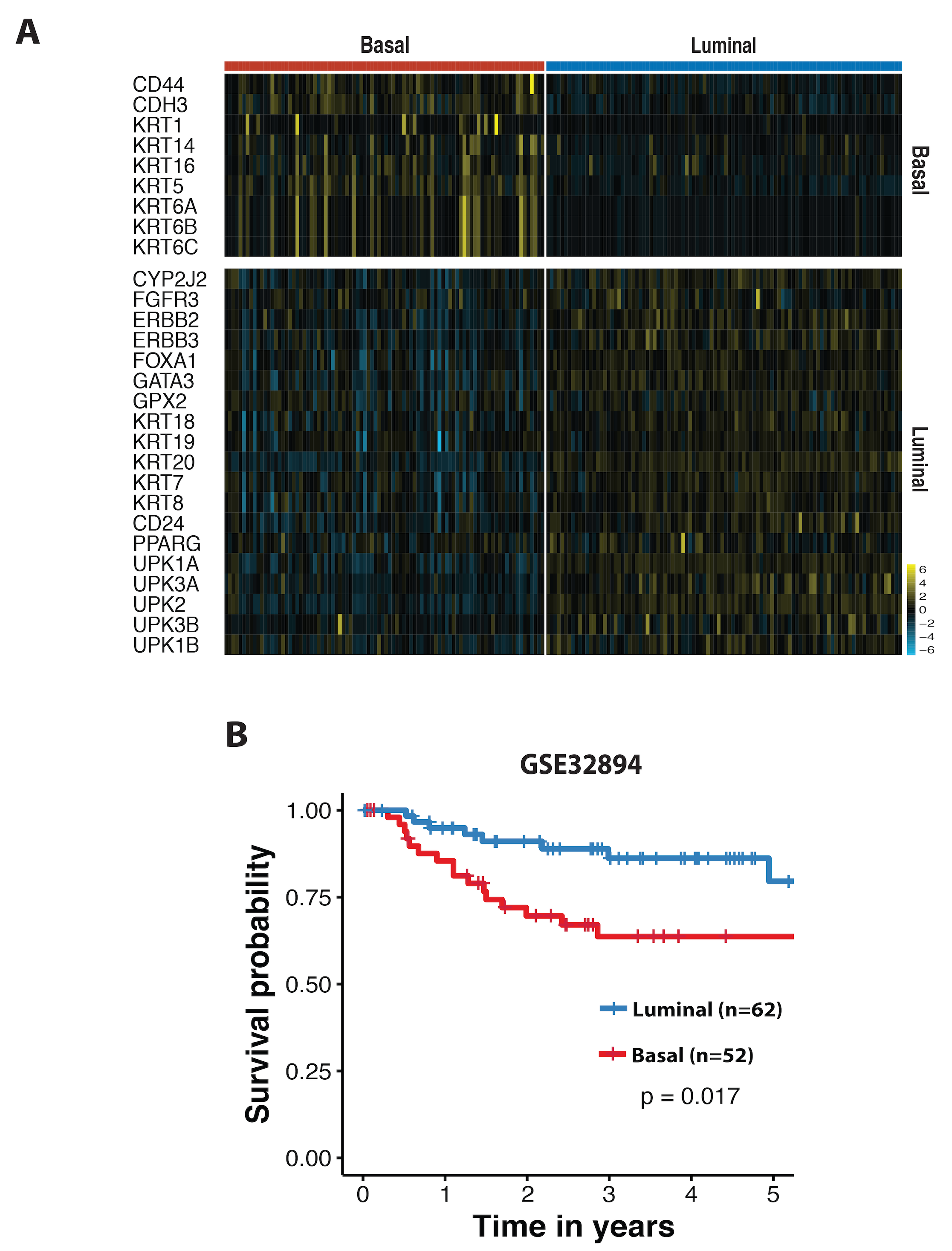

Supplement: FIGURE S4 — Heatmap and K–M plots for basal and luminal subtypes of GSE32894. (A) Heatmap depicts the expression profiles of basal (up) and luminal (down) biomarkers in GSE32894. The yellow and turquoise color corresponds to high and low relative expression, respectively. B. A K-M plot for the overall 5-year survival of basal and luminal subtypes (basal = 52, luminal = 62, p < 0.01). [file Image_4.TIF]

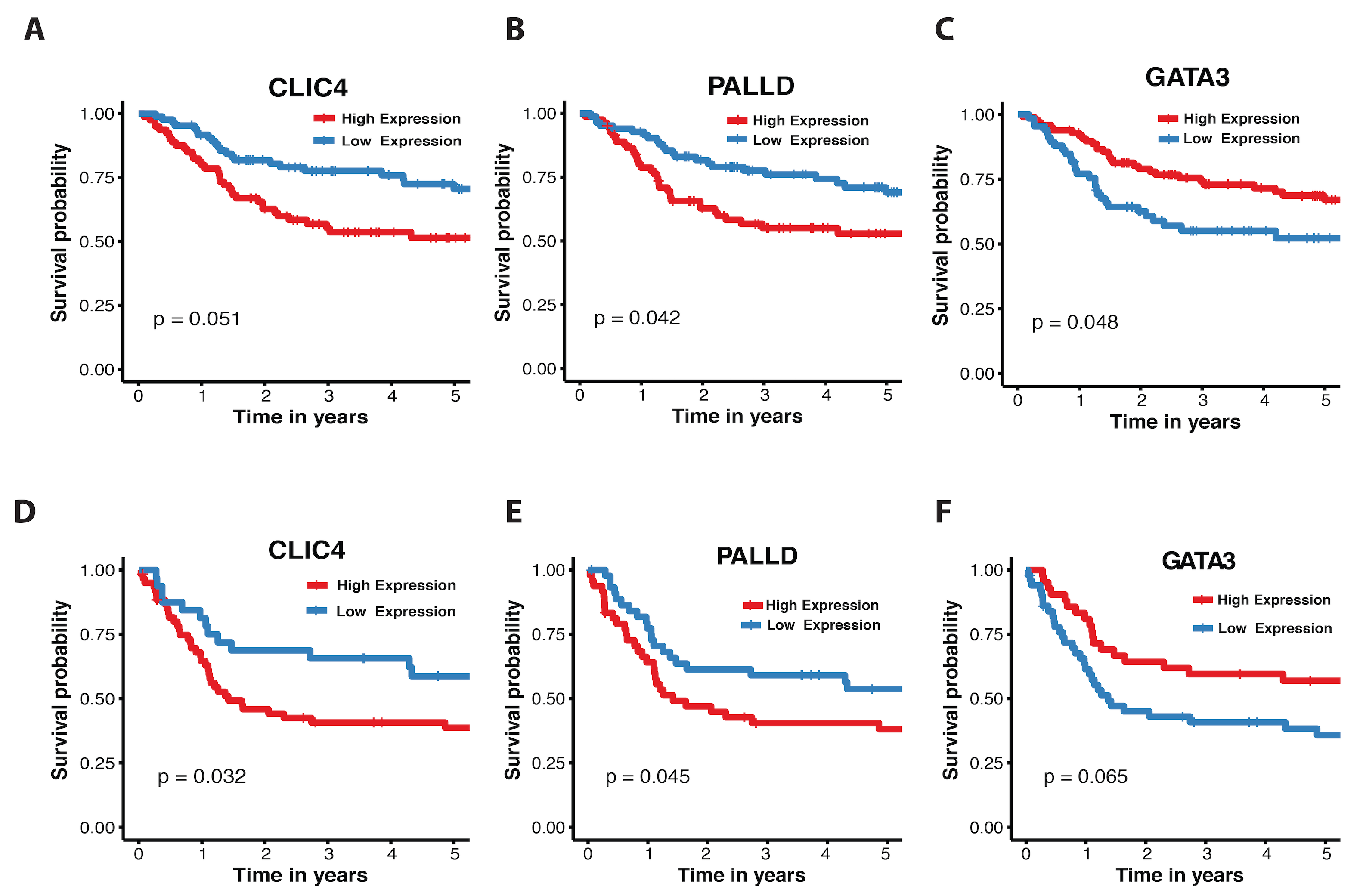

Supplement: FIGURE S5 — Kaplan-Meier plots for CLIC4, PALLD, and GATA3 in GSE13507 and GSE31684. (A–C) K–M survival curves showing overall survival according to high expression and low expression of CLIC4, PALLD, and GATA3 in GSE13507. (D–F) K–M survival curves showing overall survival according to high expression and low expression of CLIC4, PALLD, GATA3, and MIR100HG in GSE31684. [file Image_5.TIF]
