# Supplementary material for: Identification of miR-200c and miR141-Mediated lncRNA-mRNA Crosstalks in Muscle-Invasive Bladder Cancer Subtypes
Source: Front Genet. 2018 Sep 28;9:422. doi: 10.3389/fgene.2018.00422 (PMC6172409; doi:10.3389/fgene.2018.00422)
Supplement: Supplementary file 6 [file Table_1.docx]

**Supplementary Table 1. Correlation between clinical characteristics and MIBC subtypes.**

| **Variables** | **Cohort** | ***P-*value^*^** |
| --- | --- | --- |
| **Subtype**  Basal  luminal  **Stage**  I  II  III  IV  Not Available  **Age** | 210  193  2  129  138  132  2 | <0.001  0.4498 |
| 34$\leq$59  60$\leq$68  69$\leq$75  76$\leq$90  **Gender**  Male  Female  **Smoking history**  Smoker  Non-smoker  **Status**  Alive  Dead  **Histology**  Pillary  Non-pillary  Not Available | 87  112  95  109  297  106  294  109  298  105  128  270  5 | 0.0612  0.3345  0.0173  <0.001 |
| **Grade**  High grade  Low grade  Not Available | 380  20  3 | <0.001 |

Notes：*χ^2^ test.
